# Concurrent chemoradiotherapy with raltitrexed and nedaplatin regimen for esophageal squamous cell carcinoma

**DOI:** 10.1097/MD.0000000000018732

**Published:** 2020-01-24

**Authors:** Xiangnan Qiu, Jing Li, Han Zhou, Meng Zhang, Changchen Jiang, Zetian Shen, XiXu Zhu, Aomei Li, Yuxin Che, Tiancong Wu, Zhen Wang

**Affiliations:** Department of Radiation Oncology, Jinling Hospital, Medical School of Nanjing University, Nanjing, PR China.

**Keywords:** chemoradiotherapy, esophageal cancer, nedaplatin, raltitrexed

## Abstract

**Background:**

The aim of the study reported here was to evaluate the feasibility and safety of **raltitrexed and nedaplatin** with concurrent radiotherapy in patients with unresectable, locally advanced esophageal squamous cell carcinoma (ESCC).

**Methods:**

Eligible patients were adults with newly diagnosed untreated, unresectable esophageal cancer in stages I to IV with lymph node metastases or cervical esophageal cancer. Patients received nedaplatin 25 mg/m^2^ per day on day 1–3, raltitrexed 3 mg/m^2^ on days 1 repeated every 21 days for 2 cycles, and combined concurrent radiotherapy (2 Gy/fraction, total dose of 60 Gy).

**Result:**

Thirty patients were included with squamous cell carcinoma. The median follow-up duration was 24 months. The overall response rate was 90%. The 1-year and 2-year overall survival rates for all patients were 70.4% and 55.7% with a median survival time of 30 months, and the median progression free survival was 20 month. The major toxicities were leukopenia and thrombopenia, with grade 3 to 4 leukopenia and thrombopenia were 50% and 30% of patients.

**Conclusion:**

Concurrent chemoradiotherapy with raltitrexed and nedaplatin agents frequently caused myelosuppression but was highly active and suggested to be a promising treatment option for locally advanced ESCC.

## 1 Introduction

Esophageal cancer is one of the most lethal malignancies.^[1]^ About one-half of patients presented with locally advanced stage at the time of diagnosis.^[2]^ Since the publication of long-term follow-up data from the landmark RTOG 8501 clinical trial, definitive concurrent chemoradiotherapy (CCRT) with 5-Fu and cisplatin improved survival for patients with locally advanced esophageal carcinoma,^[3]^ and established it as the standard treatment for patients with unsectable, locally advanced esophageal carcinoma.^[4]^ However, 5-Fu is known to increase acute mucosal reactions, and inducing high rates of esophagitis and cardiotoxicity, and need a longer time spent receiving continuous infusion chemotherapy and longer hospital stays.^[5,6]^ Moreover, cisplatin is similarly difficult to administer due to prolonged intravenous hydration is indispensable.^[7]^ It is necessary to investigate more convenient and efficacy chemotherapy regimen for patients with unresectable, locally advanced esophageal carcinoma. Raltitrexed is a thymidylate synthase inhibitor that has anticancer effects as shown in advanced gastro-esophageal cancers.^[8]^ Additionally, a recently published study has reported raltitrexed could significantly enhance the radiosensitivity of esophageal squamous cell carcinoma (ESCC) cells with increased DNA double-strand breaks, the G2/M arrest, and the apoptosis of ESCC cells induced by radiation.^[9]^ Nedaplatin is a derivate of platinum that shows anti-tumor activity similar to that of cisplatin and has less renal and gastrointestinal toxicity.^[7,10]^ In patients with metastatic/recurrent or advanced ESCC, nedaplatin-based regimens had comparable efficacy, less toxicity and improved tolerability compared with cisplatin-based regimens.^[10]^ Raltitrexed and nedaplatin does not need continuous infusion and intravenous hydration, respectively, and easier to administer. These previous studies suggested that the combination of raltitrexed plus nedaplatin may be an efficacy regimen in CCRT for patients with unresectable, locally advanced esophageal carcinoma. Therefore, in this retrospective study, we evaluated the feasibility and safety of raltitrexed plus nedaplatin administered concurrently with radiotherapy in patients with unresectable, locally advanced esophageal carcinoma.

## 2 Materials and methods

### 2.1 Patients

Patients with ESCC were retrospectively collected between August 2015 and March 2017. Unresectable patients were defined as patients with locally unresectable carcinoma of the esophagus (T4N0-N M0), a cervical carcinoma of the esophagus or patients with involvement of celiac or supraclavicular lymph nodes (M1a); the inclusion criteria were:

1. Histologically confirmed SCC;
2. patients with locally unresectable carcinoma of the esophagus (T4N0–1 M0), a cervical carcinoma of the esophagus or patients with involvement of celiac or supraclavicular lymph nodes (M1a);
3. All patients have not received previous CCRT treatment.
4. Eastern Cooperative Oncology Group performance status scores (ECOG) of 0-2.

Patients with an esophageal perforation, esophageal fistula, tumor bleeding, distant organ metastases, serious complications, severe infection, or mental disorder, were excluded from the study. Written informed consent was obtained from all patients prior to enrolment. Tumor length was measured by esophagography before treatment. All the patients were evaluated before treatment by the following: physical examination, upper digestive endoscopy, upper gastrointestinal radiography, cervical ultrasound, and cervical/chest/abdomen computed tomography (CT) scan. Positron emission tomography-CT scan (PET-CT) was not essential. Tumor response was assessed using cervical/chest/abdomen CT scan, and upper gastrointestinal radiography. Myocardial zymogram examination and electrocardiography were used to detect treatment-induced heart damage.

### 2.2 Treatments

Intensity-modulated radiation therapy with a 6-MV X-ray was used to deliver a total dose of 60 Gy (1.8–2.0 Gy per fraction) to the primary tumor and 50 Gy to the subclinical region. During treatment, verification images were performed weekly. Gross tumor volume was defined as the total volume of the primary tumor (GTV) and involved lymph nodes (GTVnd). The clinical tumor volume (CTV) was delineated as GTV plus 3 to 4 cm and GTVnd plus 1 to 2 cm margins in the vertical direction, which covered the corresponding lymphatic drainage areas. Planning tumor volume (PTV) was defined as CTV plus 5 mm margins in all directions. Based on the dose-volume histogram, the organ dose limits were set as follows: Mean lung dose ≤16 Gy, V20 ≤30%; mean heart dose ≤40 Gy; and maximum spinal cord dose ≤45 Gy.

Chemotherapy consisted of 3 mg/m^2^ raltitrexed given on days 1 and 22 combined with 80 mg/m^2^ nedaplatin given on days 1 to 3 and 22 to 24. The chemotherapy dose was reduced by 20% in the subsequent cycle if grade 4 myelotoxicity or grade ≥3 non-myelotoxicity toxicity occurred, and chemotherapy and radiotherapy were suspended until bone marrow/other organ functions normalized.

### 2.3 Trial end points

The primary trial end point was the overall response rate (ORR) evaluated 6 weeks after the end of the treatment.^[11]^ The ORR (complete remission + partial remission) was based on Response Evaluation Criteria in Solid Tumors version 1.1 (RECIST 1.1).^[12]^ Secondary end points included the overall survival rates (OS), the progression free survival (PFS) and therapy-related adverse reactions. OS was defined as the length of time from the start of treatment until death from any cause, censoring, or the last follow-up visit.^[13]^ PFS was defined as the length of time from the start of treatment until disease progression/recurrence, death from any cause, or the last follow-up visit; acute adverse reactions include haematological and nonhematological toxicity were evaluated according to Common Terminology Criteria for Adverse Events version 3.0.^[14]^ Patterns of failure were defined as the first site of failure. Locoregional failure included the primary tumor and regional lymph nodes. Distant failure included any site beyond the primary tumor and regional lymph nodes.

### 2.4 Follow-up

The initial follow-up visit was scheduled 6 weeks after the end of treatment, with subsequent follow-up visits taking place every 3 months during the first year. Thereafter, if disease remained stable, patients were followed up once every 6 months for 3 years, and subsequently once every year. The follow-up schedule was designed to detect any delayed side effects, and to establish final treatment outcomes. Follow-up evaluations included an assessment of signs and symptoms, Karnofsky Performance Scale score, routine blood tests, tumor markers, and imaging examinations, such as cervical/abdominal ultrasound, upper gastrointestinal radiography, cervical/chest CT, and PET-CT when available.

### 2.5 Statistical methods

Assuming a drop-out rate, we calculated the required total sample size as 30 patients. SPSS version 22.0 (IBM, Armonk, NY) was used for statistical analysis. The continuous variables were expressed as median (interquartile range) and the categorical variables as frequencies and percentages. The PFS and OS curves were estimated by Kaplan–Meier analysis. A two-sided *P* value of, .05 was considered significant.

## 3 Result

### 3.1 Patient characteristics

Baseline characteristics of all 30 patients are listed in Table 1. The median age was 68.5 years. 80% of patients were male. All treated patients had an ECOG of 0 or 1. Median tumor length was 5.0 cm (range, 1–11 cm). 29 patients completed the radiotherapy with median dose was 60 Gy, 1 patients had interruption of treatment when received 36 Gy due to esophageal fistula. 26 patients completed the chemotherapy as planned. The second nedaplatin dose was reduced by 25% in two patient due to grade 4 myelotoxicity occurred. 2 patients received 1 cycle of concurrent chemotherapy only, 1 patients because of grade 4 myelotoxicity occurred, 1 patients appeared esophageal fistula. The rate of completion of this regimen was 86.7%.

**Table 1:**
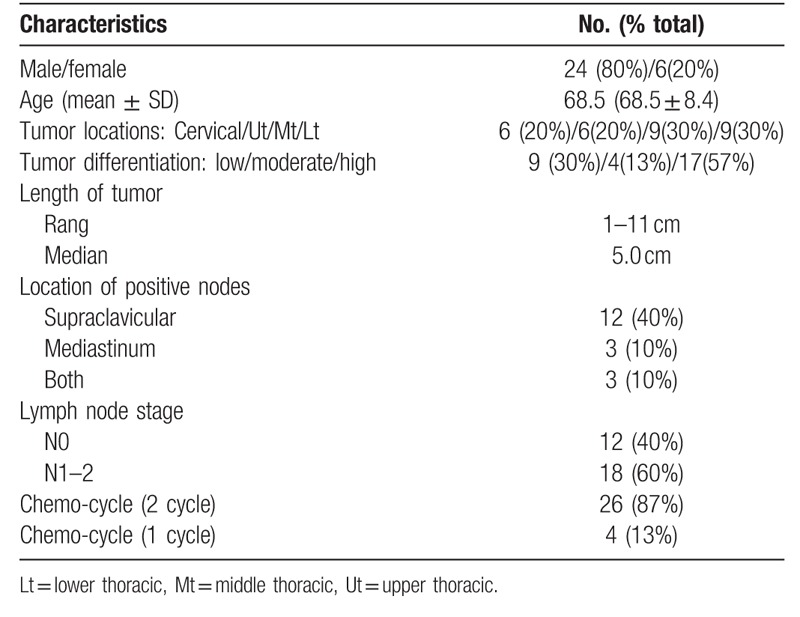
Baseline characteristics of patients.

### 3.2 Efficacy outcomes

**Figure 1:**
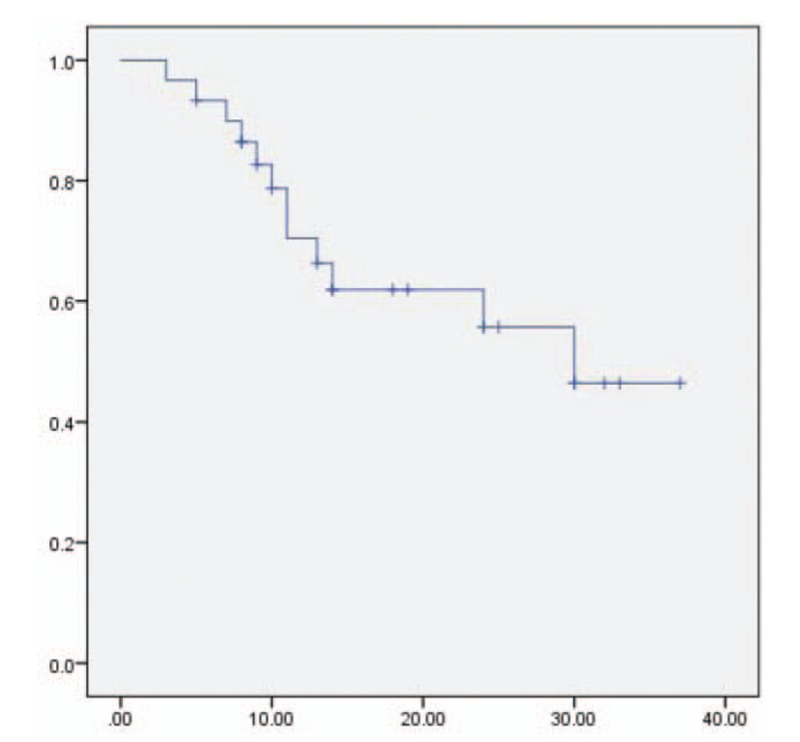
Kaplan–Meier survival curves of overall survival (OS) time for patients stratified by treatment with raltitrexed and nedaplatin.

All patients were evaluated for treatment response 6 weeks after completion of treatment. Notable, ORR was up to 90%. For surviving patients, the median follow-up time was 24 months (range, 19–29.5 m). Total median OS was 30 months and the 1- and 2-year OS rates in all patients were 70.4% and 55.7%. The median PFS was 20 months, with the 1- and 2-year PFS rates were 74.8% and 43.3% (Fig. 1).

### 3.3 Patterns of failure

A total of 13 (43.3%) patients had loco-regional or distant treatment failure, first site of treatment failure loco-regional in 8 patients (61.5%) and first site of treatment failure was distant in 5 patients (38.5%).

### 3.4 Adverse events associated with CCRT

The major toxicities were leukopenia and thrombopenia. At least III° leukopenia and thrombopenia were seen in 50% and 30% of patients. Other toxicities of grade ≥3 included oesophagitis (one patient) and pain in upper limb (one patient). No grade ≥3 anaemia and cardiotoxicity were observed. One patients developed esophageal fistula at a radiation dose of 36 Gy with 1 cycle concurrent raltitrexed/nedaplatin chemotherapy. There was no treatment-related death and radiation-induced lung injury.

## 4 Discussion

In this present study, raltitrexed/nedaplatin was associated with a high ORR rate (90%), prolonged PFS (median: 20 months), prolonged OS (MST: 30 months, 1- and 2-year survival rate: 70.4%, 55.7%), and relatively good feasibility in patients with unresectable, advanced locally esophageal cancer. Major treatment related toxicity was related to myelosuppression, but almost myelosuppression was controllable and transitory, and the rate of completion of this regimen was high (86.7%).

An overview of different studies evaluating ORR, mPFS, median survival time, and overall survival of different CCRT regimens for ESCC is shown in Table 2. The complete response of the primary tumor, was difficult to assess because RECIST 1.1 guidelines do not refer to endoscopy criteria in much detail. CT scan is still viewed as an appropriate method to assess response, but confirmation of the disappearance of the esophageal tumor by CT scan after chemoradiation is not possible because of residual thickening of the esophageal wall. Owing to these difficulties to confirm complete response, we assessed the primary tumor with CT scan and classified complete response merge into partial response. Compared with previous studies, the ORR in our study was favorable, especially higher than results in FP-based concurrent CCRT by Conroy et al (65%) and JCOG9516 study (68.3%) for patients with unresectable, advanced esophageal cancer.^[15,16]^

**Table 2:**
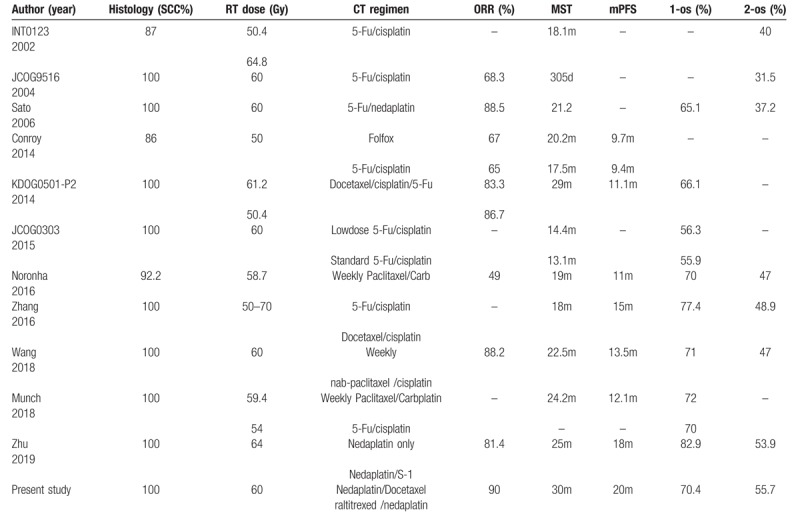
Outcomes of previous studies for chemoradiotherapy for the treatment of esophageal squamous cell carcinoma.

In those studies of Table 2, the survival showed great variation, with the MST ranging from 305 days to 29 months, the 1-year OS ranging from 52.4% to 82.9%, and the 2-year OS ranging from 15% to 51.3%. In our study, the 1-year and 2-year OS rates were 70.4% and 55.7%, respectively, with a median survival time of 30 months for all patients.^[5,7,13–15,17–22]^ The clinical outcome in our study compares generally favorable with those studies of Table 2, include the fluorouracil and cisplatin group in the RTOG 85–01 trial, INT0123 trial, and JCOG0303 trial.^[3,5,16]^ Although our results should be interpreted with caution for the small sample size and short observation period, median survival time of 30 months and 2-year OS rate of 55.7% can be looked as encouraging, indicating the efficacy of weekly schedule of raltitrexed/nedaplatin agents for unresectable, advanced esophageal cancer.

Among those studies, one which we would like to focus on was the study reported by Zhu et al.^[22]^ In this study, a total of 70 patients were treated for ESCC with radiotherapy (median dose 64 Gy, range, 60–66 Gy) combined with concurrent chemotherapy (27 patients with NDP/S-1 regimen, 30 patients with NDP/docetaxel regimen, and 13 patients with NDP alone regimen). The overall response rate was 81.4%. The 1-year and 2-year OS rate was 82.9% and 53.9%, respectively, with a median survival time of 25 months. Compared with this result, the overall response rate and median survival time in our study was better, while the 1-year OS was much lower (70.4% vs 82.9%). A possible explanation was the differences in radiation dose, radiation volume and chemotherapy regimens. The radiation dose of our study was lower than that of Zhu's study, with a median dose of 60 Gy vs 64 Gy (range, 60–66 Gy). Although the optimal radiation dose has not been established for ESCC, some studies demonstrated that a high radiation dose might yield better prognosis. Nayan et al reported that high-dose radiotherapy (64.8 Gy) with concurrent chemotherapy seems to be more effective with acceptable toxicity.^[23]^ In our study, all patients received a total radiation dose of 60 Gy, and no patient received more than 60 Gy, which might partially attribute to the relatively dismal outcome compared with that in Zhu's study. Another reason was that almost patients in Zhu's study received NDP/docetaxel or NDP/S-1 regimen concurrently with radiotherapy. Although no sufficient evidence has been established for NDP/docetaxel or NDP/S-1 regimen in treatment of ESCC, several studies have reported promising outcomes for S-1 in the treatment of gastrointestinal tumors.^[24,25]^ Additionally, two studies observed good anti-tumor effects and sensitization of radiotherapy in patients with EC when S-1 was used in multi-drug chemoradiotherapy.^[26,27]^ Regimens combining docetaxel with platinum-based drugs are extensively used for numerous types of solid malignant tumors. A regimen combining docetaxel, NDP and 5-FU was identified to be effective for ESCC.^[28–30]^ The adoption of NDP/docetaxel or NDP/S-1 regimen as concurrent chemotherapy might contribute to a favorable survival in Zhu's study and need to be further evaluated.

In our study, a total of 41.7% of patients had loco-regional or distant treatment failure, which is lower than data from INT0123 and Munch et al, in which EC patients were treated with CCRT with cisplatin /5FU.^[13,16]^ However, in that studies median follow-up was longer than in our study, which might explain the higher rate of loco-regional recurrences or distant treatment failure. The initial site(s) of failure were predominantly locoregional in nature, with 54.5% of patients experiencing failure within or at the margin of the treatment field as some component of initial failure, which is line with data from Ruppert et al and KDOG0501-P2.^[17,31]^

As shown in our study, the most frequent acute toxicity was leukopenia and thrombopenia. Although the leukopenia and thrombopenia were slightly higher than several studies, our results were comparable to results by Yamashita et al and Munch et al.^[13,32]^ In Yamashita et al analysis, patients with locally advanced ESCC were treated with 50.4 Gy and concomitant chemotherapy with nedaplatin/5FU, ≥ III° leucopenia was seen in 62% patients, which is higher than our result (50%), and ≥ III° thrombopenia (27%) was in line with our result (30%).^[32]^ In addition, compared to the results presented by Münch et al, the rate of ≥ III° thrombopenia (48%) was comparable to our data (50%) in 5-FU and cisplatin group treated with CCRT of ESCC.^[13]^ It seems likely raltitrexed/nedaplatin regimen lead to more leucopenia and thrombopenia, but the ≥ III°leucopenia and thrombopenia in our study was controllable and transitory, and most patients were therefore able to complete the regimen without suspension of treatment or reduction of dose in the second cycle of chemotherapy. Therefore, the rate of completion of this regimen was high (86.7%), which was higher than data in JCOG9516 trail (77%) and in standard dose 5-Fu/cisplatin group in JCOG0303 trail (82%).^[5,15]^ However, the incidence of grade ≥3 gastrointestinal toxicity, hyponatremia, and oesophagitis were relatively low in our study, compared with the previous studies. Notable, no patient had cardiac toxicity of grade 1 or higher in the present protocol. Larger studies suggest an incidence of symptomatic cardiotoxicity of 1.2% to 4.3% during 5-Fu treatment, however subclinical cardiac influence are common. Possible risk factors are cardiac comorbidity, continuous infusion schedules and concomitant cisplatin treatment.^[6]^ It was reported that the approach of switching from 5-Fu/capecitabine to raltitrexed for patients with 5-FU cardiotoxicity is safe and offers the lowest risk of recurrent cardiotoxicity.^[33]^ Fistula formation is caused by CRT during or after the treatment and can be the cause of treatment related death. In JCOG 0303, esophageal fistula associated with CCRT developed in 22% of the patients.^[5]^ In this study, esophageal fistula was observed in one patient during CRT.

This study has a few limitations that need to be considered when interpreting the results. The study is limited by its retrospective nature, and we cannot account for potential selection bias, which may limit the generalizability of our results. Second, our sample size is small, and further independent studies in larger populations are needed to confirm and validate our results. Finally, it is possible that treatment related toxicities were underestimated due to the study's retrospective setting. Furthermore, a clinical complete response is more typically defined as a negative biopsy at esophagoscopy. However, in the previous study, we observed patients’ refusal to undergo esophagoscopy; hence, we defined complete response on a CT scan or esophagoscopy.

In conclusion, the present study suggests that CCRT with raltitrexed and nedaplatin agents frequently caused myelosuppression but was highly active and suggested to be a promising treatment option for locally advanced ESCC. These results suggest raltitrexed/nedaplatin could be used as an alternative for cisplatin/5-FU in CCRT for EC patients which should be further evaluated.

## Author contributions

**Conceptualization:** Zhen Wang.

**Data curation:** Jing Li, Tiancong Wu.

**Formal analysis:** Han Zhou.

**Investigation:** Meng Zhang.

**Methodology:** Changchen Jiang.

**Project administration:** Zetian Shen.

**Resources:** XiXu Zhu.

**Supervision:** Aomei Li.

**Validation:** Yuxin Che.

**Writing – original draft:** Xiangnan Qiu.

**Writing – review & editing:** Tiancong Wu.

## References

[1] Torre LA Siegel RL Ward EM Global cancer incidence and mortality rates and trends—an update. Cancer Epidemiol Biomarkers Prev 2016;25:16–27. 26667886 10.1158/1055-9965.EPI-15-0578

[2] Shahbaz SCM Luketich JD Landreneau RJ Esophageal cancer: an update. Int J Surg 2010;8:417–22. 20601255 10.1016/j.ijsu.2010.06.011

[3] Cooper JS Guo MD Herskovic A Chemoradiotherapy of locally advanced esophageal cancer: long-term follow-up of a prospective randomized trial (RTOG 85-01). Radiation Therapy Oncology Group. JAMA 1999;281:1623–7. 10235156 10.1001/jama.281.17.1623

[4] Lordick F Mariette C Haustermans K Oesophageal cancer: ESMO Clinical Practice Guidelines for diagnosis, treatment and follow-up. Ann Oncol 2016;27:v50–7. 27664261 10.1093/annonc/mdw329

[5] Shinoda M Ando N Kato K Randomized study of low-dose versus standard-dose chemoradiotherapy for unresectable esophageal squamous cell carcinoma (JCOG0303). Cancer Sci 2015;106:407–12. 25640628 10.1111/cas.12622 PMC4409884

[6] Polk A Vaage-Nilsen M Vistisen K Cardiotoxicity in cancer patients treated with 5-fluorouracil or capecitabine: a systematic review of incidence, manifestations and predisposing factors. Cancer Treat Rev 2013;39:974–84. 23582737 10.1016/j.ctrv.2013.03.005

[7] Sato Y Takayama T Sagawa T A phase I/II study of nedaplatin and 5-fluorouracil with concurrent radiotherapy in patients with esophageal cancer. Cancer Chemother Pharmacol 2006;58:570–6. 16463059 10.1007/s00280-006-0193-x

[8] Eatock MM Anthony DA El-Abassi M A dose-finding study of raltitrexed (tomudex) with cisplatin and epirubicin in advanced gastro-oesophageal adenocarcinoma. Br J Cancer 2000;82:1925–31. 10864199 10.1054/bjoc.2000.1165 PMC2363246

[9] Ding WX Liu S Ma JX Raltitrexed increases radiation sensitivity of esophageal squamous carcinoma cells. Cancer Cell Int 2019;18:36. 10.1186/s12935-019-0752-y PMC6378748 30820189

[10] Zhang F Wang Y Wang ZQ Efficacy and safety of cisplatin-based versus nedaplatin-based regimens for the treatment of metastatic/recurrent and advanced esophageal squamous cell carcinoma: a systematic review and meta-analysis. Dis Esophagus 2017;30:1–8. 10.1111/dote.12490 27868295

[11] Servagi-Vernat S Créhange G Roullet B Phase II study of a nedaplatin-based adapted chemotherapy regimen combined with radiotherapy in patients 75 years and older with esophageal cancer. Drugs Aging 2015;32:487–93. 26038198 10.1007/s40266-015-0275-8

[12] Eisenhauer EA Therasse P Bogaerts J New response evaluation criteria in solid tumours: revised RECIST guideline (version 1.1). Eur J Cancer 2009;45:228–47. 19097774 10.1016/j.ejca.2008.10.026

[13] Wang D Zhang W Qian D Efficacy and safety of weekly nab-paclitaxel plus cisplatin with concurrent intensity-modulated radiotherapy in patients with inoperable, locally advanced esophageal cancer: a pilot trial. Onco Targets Ther 2018;28:6333–8. 10.2147/OTT.S168275 PMC6167982 30319273

[14] Trotti A Colevas AD Setser A CTCAE v3.0: development of a comprehensive grading system for the adverse effects of cancer treatment. Semin Radiat Oncol 2003;13:176–81. 12903007 10.1016/S1053-4296(03)00031-6

[15] Conroy T Galais MP Raoul JL Definitive chemoradiotherapy with FOLFOX versus fluorouracil and cisplatin in patients with oesophageal cancer (PRODIGE5/ACCORD17): final results of a randomised, phase 2/3 trial. Lancet Oncol 2014;15:305–14. 24556041 10.1016/S1470-2045(14)70028-2

[16] Ishida K Ando N Yamamoto S Phase II study of cisplatin and 5-fluorouracil with concurrent radiotherapy in advanced squamous cell carcinoma of the esophagus: a Japan Esophageal Oncology Group (JEOG)/Japan Clinical Oncology Group trial (JCOG9516). Jpn J Clin Oncol 2004;34:615–9. 15591460 10.1093/jjco/hyh107

[17] Minsky BD Pajak TF Ginsberg RJ INT 0123 (Radiation Therapy Oncology Group 94-95) phase III trial of combined-modality therapy for esophageal cancer: high-dose versus standard-dose radiation therapy. J Clin Oncol 2002;20:1167–74. 11870157 10.1200/JCO.2002.20.5.1167

[18] Higuchi K Komori S Tanabe S Definitive chemoradiation therapy with docetaxel, cisplatin, and 5-fluorouracil (DCF-R) in advanced esophageal cancer: a phase 2 trial (KDOG 0501-P2). Int J Radiat Oncol Biol Phys 2014;89:872–9. 24867539 10.1016/j.ijrobp.2014.03.030

[19] Noronha V Prabhash K Joshi A Clinical outcome in definitive concurrent chemoradiation with weekly paclitaxel and carboplatin for locally advanced esophageal and junctional cancer. Oncol Res 2016;23:183–95. 27053347 10.3727/096504016X14537290676865 PMC7838643

[20] Zhang P Xi M Li QQ Concurrent cisplatin and 5-fluorouracil versus concurrent cisplatin and docetaxel with radiotherapy for esophageal squamous cell carcinoma: a propensity score-matched analysis. Oncotarget 2016;7:44686–94. 27183916 10.18632/oncotarget.9301 PMC5190128

[21] Münch S Pigorsch SU Devečka M Comparison of definite chemoradiation therapy with carboplatin/paclitaxel or cisplatin/5-fluoruracil in patients with squamous cell carcinoma of the esophagus. Radiat Oncol 2018;13:139. 30068371 10.1186/s13014-018-1085-z PMC6090949

[22] Zhu H Ge X Lu Y Nedaplatin-based chemotherapy regimens combined with concurrent radiotherapy as first-line treatment for stage II-III esophageal squamous cell carcinoma. Oncol Lett 2019;17:594–602. 30655806 10.3892/ol.2018.9564 PMC6313161

[23] Nayan N Bhattacharyya M Jagtap VK Standard-dose versus high-dose radiotherapy with concurrent chemotherapy in esophageal cancer: a prospective randomized study. South Asian J Cancer 2018;7:27–30. 29600230 10.4103/sajc.sajc_178_17 PMC5865091

[24] Koizumi W Kurihara M Nakano S Phase II study of S-1, a novel oral derivative of 5-fluorouracil, in advanced gastric cancer. For the S-1 Cooperative Gastric Cancer Study Group. Oncology 2000;58:191–7. 10765119 10.1159/000012099

[25] Nakata B Mitachi Y Tsuji A Combination phase I trial of a novel oral fluorouracil derivative S-1 with low-dose cisplatin for unresectable and recurrent gastric cancer (JFMC27-9902). Clin Cancer Res 2004;10:1664–9. 15014017 10.1158/1078-0432.ccr-03-0045

[26] Tanaka Y Yoshida K Tanahashi T Phase II trial of neoadjuvant chemotherapy with docetaxel, nedaplatin, and S1 for advanced esophageal squamous cell carcinoma. Cancer Sci 2016;107:764–72. 27061001 10.1111/cas.12943 PMC4968606

[27] Tanaka Y Yoshida K Osada S Docetaxel, nedaplatin, and S-1 (DGS) chemotherapy for advanced esophageal carcinoma: a phase I dose-escalation study. Anticancer Res 2011;31:4589–97. 22199335

[28] Guo JF Zhang B Wu F A phase II trial of docetaxel plus nedaplatin and 5-fluorouracil in treating advanced esophageal carcinoma. Chin J Cancer 2010;29:321–4. 20193118 10.5732/cjc.009.10432

[29] Akutsu Y Shuto K Kono T A phase 1/11 study of second-line chemotherapy with fractionated docetaxel and nedaplatin for 5-FU/cisplatin-resistant esophageal squamous cell carcinoma. Hepatogastroenterology 2012;59:2095–8. 22328303 10.5754/hge11952

[30] Miyazaki T Ojima H Fukuchi M Phase II study of docetaxel, nedaplatin, and 5-fluorouracil combined chemotherapy for advanced esophageal cancer. Ann Surg Oncol 2015;22:3653–8. 25691281 10.1245/s10434-015-4440-4

[31] Ruppert BN Watkins JM Shirai K Cisplatin/Irinotecan versus carboplatin/paclitaxel as definitive chemoradiotherapy for locoregionally advanced esophageal cancer. Am J Clin Oncol 2010;33:346–52. 19841574 10.1097/COC.0b013e3181aaca26

[32] Yamashita H Omori M Takenaka R Involved-field irradiation concurrently combined with nedaplatin/5-fluorouracil for inoperable esophageal cancer on basis of [18]FDG-PET scans: a phase II study. Radiother Oncol 2014;113:182–7. 25466372 10.1016/j.radonc.2014.11.004

[33] Ransom D Wilson K Fournier M Final results of Australasian Gastrointestinal Trials Group ARCTIC study: an audit of raltitrexed for patients with cardiac toxicity induced by fluoropyrimidines. Ann Oncol 2014;25:117–21. 24299960 10.1093/annonc/mdt479

